# 70-Gene signature-guided adjuvant systemic treatment adjustments in early-stage ER+ breast cancer patients: 7-year follow-up of a prospective multicenter cohort study

**DOI:** 10.1007/s10549-024-07496-3

**Published:** 2024-09-30

**Authors:** Eline E. F. Verreck, Anne Kuijer, Julia E. C. van Steenhoven, José H. Volders, Annette W. G. van der Velden, Sabine Siesling, Anja N. H. Timmer-Bonte, Tineke J. Smilde, Alex L. T. Imholz, Charlotte F. J. M. Blanken-Peeters, Bart de Valk, Suzan Vrijaldenhoven, Willem B. Lastdrager, Annebeth W. Haringhuizen, Jarmo C. B. Hunting, Sjoerd Hovenga, Peter Nieboer, Hanneke M. Zuetenhorst, Geert W. M. Tetteroo, Carolien H. Smorenburg, Marissa C. van Maaren, Thijs van Dalen

**Affiliations:** 1 University Utrecht, Utrecht, the Netherlands; 2 Department of Surgery, Diakonessenhuis, Bosboomstraat 1, 3582 KE Utrecht, the Netherlands; 3 Department of Surgery, St. Antonius Hospital, Nieuwegein, the Netherlands; 4 Department of Pathology, University Medical Centre Utrecht, Utrecht, the Netherlands; 5 Department Internal Medicine, Martini Hospital, Groningen, the Netherlands; 6 Department of Health Technology and Services Research, Technical Medical Centre, University of Twente, Enschede, the Netherlands; 7 Department of Research and Development, Netherlands Comprehensive Cancer Organisation (IKNL), Utrecht, the Netherlands; 8 Department Internal Medicine, Alexander Monro Hospital, Bilthoven, the Netherlands; 9 Department Internal Medicine, Jeroen Bosch Hospital, Den Bosch, the Netherlands; 10 Department Internal Medicine, Deventer Hospital, Deventer, the Netherlands; 11 Department of Surgery, Rijnstate Hospital Arnhem, Arnhem, the Netherlands; 12 Deparment Internal Medicine, Spaarne Gasthuis, Hoofddorp, the Netherlands; 13 Department Internal Medicine, Noordwest Ziekenhuisgroep, Alkmaar, the Netherlands; 14 Department of Surgery, Gelre Hospital, Apeldoorn, the Netherlands; 15 Deparmtent Internal Medicine, Gelderse Vallei Hospital, Ede, the Netherlands; 16 Department Internal Medicine, St. Antonius Hospital, Nieuwegein, the Netherlands; 17 Department Internal Medicine, Nij Smellinghe Hospital, Drachten, the Netherlands; 18 Deparment Internal Medicine, Wilhemina Hospital, Assen, the Netherlands; 19 Deparment Internal Medicine, Franciscus Gasthuis, Rotterdam, the Netherlands; 20 Department of Surgery, Ijsselland Hospital, Cappele Aan de Ijssel, the Netherlands; 21 Department Internal Medicine, Antoni Van Leeuwenhoek Hospital, Amsterdam, the Netherlands; 22 Department of Surgery, Erasmus Medical Centre, Dr. Molewaterplein 40, 3015 GD Rotterdam, the Netherlands; 23 Department of Surgery, University Medical Centre Utrecht, Utrecht, the Netherlands

**Keywords:** 70-Gene Signature, Chemotherapy decision, Luminal breast cancer

## Abstract

**Background:**

A previous prospective multicenter study revealed the change of the oncologists’ chemotherapy advice due to the 70-Gene signature (GS) test result in half of the estrogen receptor-positive (ER+) invasive early-stage breast cancer patients with disputable chemotherapy indication. This resulted in less patients receiving chemotherapy. This study aims to complement these results by the 7-year oncological outcomes according to the 70-GS test result and the oncologists’ pre-test advice.

**Methods:**

Patients operated for early-stage ER+ breast cancer with disputable chemotherapy indication, had been prospectively included between 2013 and 2015. Oncologists were asked whether they intended to administer adjuvant chemotherapy before deployment of the 70-GS test. Information on adjuvant systemic treatment and oncological outcome was obtained through active follow-up by data managers of the Netherlands Cancer Registry. The primary endpoint of this study was distant metastasis-free survival (DMFS) according to the genomic risk. Exploratory analyses were done to evaluate DMFS in relation to the oncologists’ pre-test advice.

**Results:**

After a median follow-up of 7 years, distant metastases were diagnosed in 23 of the 606 patients (3.8%) and 36 (5.9%) patients had died. The DMFS rate for the 357 70-GS genomic low-risk patients was 94.2% (95% CI 91.2–96.2) and 89.1% for the 249 genomic high-risk patients (95% CI 84.3–92.4). Of the low-risk patients 3% had received chemotherapy compared to 80% of the high-risk patients. For the subgroups based on the pre-test oncologists’ advice (no chemotherapy/chemotherapy/unsure) there were no clinically relevant differences in DMFS (89.8, 93.2 and 92.0%, respectively), while comparable proportions of patients had received chemotherapy.

**Conclusions:**

In patients with early-stage ER+ breast cancer with a disputable chemotherapy indication it is sensible to deploy the 70-GS to better select patients for adjuvant chemotherapy.

**Supplementary Information:**

The online version contains supplementary material available at 10.1007/s10549-024-07496-3.

## Introduction

Since the 1990s, adjuvant systemic therapy improved disease-free (DFS) and overall survival (OS) in women with early-stage breast cancer [1–3]. Patients having lymph node metastases (≥ N1a) and/or grade 3 tumors were candidates to receive adjuvant systemic therapy initially. After that, international breast cancer treatment guidelines gradually expanded the range of patients eligible for adjuvant chemotherapy and endocrine therapy [4–6]. This is illustrated by the treatment guideline from 2012, in which patients with grade 2 tumors > 1 cm or grade 1 tumors > 2 cm were considered eligible for adjuvant systemic chemotherapy irrespective of lymph node involvement [7].

The increased knowledge of molecular tumor subtypes and the use of gene expression profiles (GEPs) have refined insight in prognosis for individual patients [8–10]. Randomized clinical trials, such as the EORTC 10041/BIG3-04 (MINDACT) and the ECOG PACCT–1 (TailorX study) studied the role of GEPs in addition to clinicopathological characteristics [11, 12]. Those studies provided evidence that omitting adjuvant chemotherapy was safe in patients who had a low-risk GEP result. In current clinical practice, GEPs are advised in patients with estrogen receptor (ER) positive (+) early-stage breast cancer [7] and a substantial proportion of such patients are consequently spared from overtreatment by chemotherapy [13].

In a previous prospective study, including ER+ early-stage breast cancer patients in whom the benefit of adjuvant chemotherapy was considered disputable based on the clinicopathological characteristics, we evaluated the clinical impact of 70-Gene Signature (70-GS) use [13]. The study was conducted between 2013 and 2015 when the results of the aforementioned RCTs were not available yet [11, 12]. Oncologists were asked for their advice regarding the need to administer adjuvant chemotherapy before the 70-GS test result was disclosed. It was observed that the 70-GS changed the oncologists’ recommendation to administer or withhold chemotherapy in 51% of the patients [13].

To gain insight in the added value of 70-GS in adjuvant chemotherapy decision making, the latter study is complemented with follow-up data on the occurrence of locoregional recurrence (LRR), distant metastasis (DM) and contralateral breast cancer (CBC) and these oncological outcomes were analyzed according to the genomic risk. In addition, the influence on outcome of the advice to administer chemotherapy prior to the 70-GS use was addressed.

## Methods

### Study population

In the previous study—an observational, prospective, multicenter cohort study—data was collected of 660 T1-2N0-1 ER+ ductal primary invasive breast cancer patients treated with upfront surgery, diagnosed between 2013 and 2015 in 33 Dutch hospitals, in whom the benefit of adjuvant chemotherapy was considered disputable based on traditional prognostic factors. All patients were entitled to receive the 70-GS test according to the Dutch breast cancer guideline of 2012 [7]. Before deployment of the 70-GS the oncologists’ pre-test advice to administer chemotherapy was registered as a proxy of how the oncologists perceived the clinical risk to develop metastatic disease. More details on the study design of the Triple A study have been described elsewhere [13].

### Data collection

All participating hospitals were asked to securely send patient numbers of the originally included patients to the Netherlands Comprehensive Cancer Organisation (IKNL). Information regarding baseline criteria and pre-test advice had been collected prospectively as part of the original study [13]. Data on the actual administration of adjuvant chemotherapy and the incidence of LRR, CBC and DM were collected from the medical records in the participating hospitals by specifically trained data managers of the IKNL and registered in the Netherlands Cancer Registry (NCR). Vital status was obtained through linkage with the Municipal Personal Records Database, which is yearly performed. The NCR contains all newly diagnosed malignancies from 1989 on [14]. It derives information from the Dutch Nationwide Pathology Databank (Palga) [15] and the National Basic Hospital Care Registration. To ensure the quality of the registered data, quality checks are performed regularly.

### Study endpoints

The primary endpoint of this study was distant metastasis-free survival (DMFS) stratified for the 70-GS risk score. DMFS was defined as the likelihood of being alive and free of DM at the end of follow-up according to the STEEP criteria [16]. Occurrence of LRR or CBC prior to the date of development of DMs or death were not censored. Follow-up started at the date of final surgery. The outcome risk estimates of the 70-GS (low versus high risk) and the oncologists’ pre-test advice (‘no chemotherapy’, ‘chemotherapy’ versus ‘unsure’) are reported with the proportions of the patients within the groups that actually received chemotherapy to provide clinical context. Secondary outcomes included DFS, defined as the likelihood of being alive without any breast cancer manifestation (including LRR, DM, CBC) at the end of follow-up, and OS. To evaluate if the oncologists’ initial advice prior to 70-GS use was associated with DM risk, an exploratory analysis was performed to determine the same oncological outcomes stratified for the oncologists’ advice to administer chemotherapy before the 70-GS test deployment.

Lastly, we explored DMFS according to the clinical risk criteria that were applied in the aforementioned EORTC 10041/BIG3-04 (MINDACT) trial [11]. In the latter study clinical risk was dichotomized based on an expected 10-year breast cancer-specific survival (BCSS) being higher or lower than 88% without adjuvant chemotherapy estimated by Adjuvant! Online [11]. DMFS was determined for the genomic risk categories within the Adjuvant! Online-based clinical risk groups to evaluate further distinction between patients who may benefit from adjuvant chemotherapy.

### Statistical analysis

Demographic and clinical characteristics of the study population were summarized in a baseline table, according to the genomic risk and the pre-test chemotherapy advice and the distribution of variables was compared by using chi-squared tests. The percentages of patients presenting with DM, LRR, CBC as first events and those who died were calculated according to the respective genomic and pre-test chemotherapy advice groups. Kaplan–Meier survival curves were constructed, and the log-rank test was used to determine whether outcomes were significantly different between the 70-GS categories, the oncologists’ pre-test advice categories and the Adjuvant! Online risk categories (DMFS only).

Concordance between the 70-GS test results, the pre-test chemotherapy advice and the Adjuvant! Online-based risk categorization was shown in a supplementary baseline table and compared by using chi-squared tests. A *p* value < 0.05 was considered to be significant. Analyses were performed using STATA version 17.0 (StataCorp, Texas).

### Ethical considerations

The study was approved by the Medical Ethical Committee (METC) NedMec in Utrecht, the Netherlands (protocol number 12-450 was registered in the clinicaltrial.gov database; NCT02209857). All patients participating in this study signed an informed consent form prior to participation [13]. The additional data collection for the present study was separately approved by the METC and all participating hospitals were additionally asked for consent.

## Results

### Patients

From a total of 660 early-stage breast cancer patients included in the previous prospective study, follow-up could be retrieved of 606 patients. Of the remaining 54 patients, patient numbers delivered by several hospitals were not complete or could not be identified in the NCR. Most of the patients were diagnosed with stage cT1 (81%), grade 2 tumors (73%) and were node-negative (84%). See Table 1.

**Table 1:** Baseline characteristics of all invasive ductal carcinoma patients categorized by the 70-GS and the oncologists’ pre-test chemotherapy advice

Characteristics	N	70-GS (*N* = 606)			Pre-test chemotherapy advice (*N* = 606)			
		Genomic low-risk (%)	Genomic high-risk (%)	*p *value^a^	No chemotherapy (%)	Chemotherapy (%)	Unsure (%)	*p *value^a^
No. of patients	606	357 (59)	249 (41)		100 (16)	259 (43)	247 (41)	
Age group (years)								
< 40	8 (1)	4 (1)	4 (2)	0.32	0 (0)	4 (2)	4 (1)	0.63
40–49	117 (19)	78 (22)	39 (16)		17 (17)	51 (20)	49 (20)	
50–59	228 (38)	133 (37)	95 (38)		37 (37)	105 (41)	86 (35)	
60–69	230 (38)	128 (36)	102 (41)		40 (40)	92 (35)	98 (40)	
70–79	22 (4)	14 (4)	8 (3)		6 (6)	6 (2)	10 (4)	
> 80	1 (0)	0 (0)	1 (0)		0 (0)	1 (0)	0 (0)	
Surgery type								
Lumpectomy	491 (81)	284 (80)	207 (83)	0.27	85 (85)	204 (79)	202 (82)	0.37
Mastectomy	115 (19)	73 (20)	42 (17)		15 (15)	55 (21)	45 (18)	
Type of axillary surgery								
ALND	6 (1)	2 (1)	4 (2)	0.22	4 (4)	1 (1)	1 (0)	0.03
SNP	572 (95)	337 (94)	235 (94)		89 (89)	247 (95)	236 (96)	
ALND+SNP	8 (1)	7 (2)	1 (0)		1 (1)	3 (1)	4 (2)	
None	20 (3)	11 (3)	9 (4)		6 (6)	8 (3)	6 (2)	
Histological grade								
Grade 1	90 (15)	68 (19)	22 (9)	< 0.001	24 (24)	29 (11)	37 (15)	0.004
Grade 2	440 (73)	267 (75)	173 (69)		71 (71)	188 (73)	181 (73)	
Grade 3	76 (12)	22 (6)	54 (22)		5 (5)	42 (16)	29 (12)	
ER status								
Negative	1 (0)	0 (0)	1 (0)	0.23	1 (1)	0 (0)	0 (0)	0.08
Positive	605 (100)	357 (100)	248 (100)		99 (99)	259 (100)	247 (100)	
PR status								
Negative	78 (13)	31 (9)	47 (19)	0.001	15 (15)	35 (14)	28 (11)	0.19
Positive	527 (87)	326 (91)	201 (81)		84 (84)	224 (86)	219 (89)	
Unknown	1 (0)	0 (0)	1 (0)		1 (1)	0 (0)	0 (0)	
HER2 status								
Negative	585 (97)	349 (98)	236 (95)	0.004	97 (97)	249 (96)	239 (97)	0.084
Positive	15 (2)	3 (1)	12 (5)		1 (1)	10 (4)	4 (2)	
Unknown	6 (1)	5 (1)	1 (0)		2 (2)	0 (0)	4 (1)	
T-stage								
T1	492 (81)	288 (81)	204 (82)	0.67	90 (90)	202 (78)	200 (81)	0.01
T2	113 (19)	68 (19)	45 (18)		9 (9)	57 (22)	47 (19)	
T3	1 (0)	1 (0)	0 (0)		1 (1)	0 (0)	0 (0)	
N-stage^b^								
N0	510 (84)	296 (83)	214 (86)	0.13	88 (88)	217 (84)	205 (83)	0.84
N1mi	55 (9)	38 (11)	17 (7)		5 (5)	24 (9)	26 (10)	
N1	36 (6)	22 (6)	14 (6)		6 (6)	16 (6)	14 (6)	
Unknown	5 (1)	1 (0)	4 (1)		1 (1)	2 (1)	2 (1)	
Adjuvant chemotherapy								
No	397 (66)	346 (97)	51 (20)	< 0.001	65 (65)	162 (63)	170 (69)	0.33
Yes	209 (34)	11 (3)	198 (80)		35 (35)	97 (37)	77 (31)	
Adjuvant hormonal therapy								
No	111 (18)	71 (20)	40 (16)	0.23	22 (22)	44 (17)	45 (18)	0.55
Yes	495 (82)	286 (80)	209 (84)		78 (78)	215 (83)	202 (82)	

*70-GS* 70 gene signature, *ALND* axillary lymph node dissection, *SNP* sentinel node procedure, *ER* estrogen receptor, *PR* progesteron receptor

^a^Chi-squared test

^b^*N0* no lymph node metastasis, *N1mi* micrometastasis, *N1* macrometastasis

Chemotherapy had been recommended in 43% and discommended in 16% of the patients before the 70-GS test was deployed, while oncologists felt unsure in the remaining 41%. The 70-GS assigned 59% and 41% of patients to the low or high genomic risk category, respectively. Chemotherapy was administered to 3% of the genomic low-risk patients and to 80% of patients in the genomic high-risk group.

Median follow-up to first event or last observation was 7.0 years (interquartile range (IQR) 6.4 years–7.5 years). In total, DMs occurred in 23 patients (3.8%) and 36 patients (5.9%) died during the follow-up period. Eleven of the 23 patients who developed DMs had subsequently died during follow-up (Table 2).

**Table 2:** A summary of the outcome events

Event	Overall	70-GS (*N* = 606)		Pre-test chemotherapy advice (*N* = 606)		
		Genomic low-risk	Genomic low-risk	No chemotherapy	Chemotherapy	Unsure
No. of patients (%)	606	357 (59)	249 (41)	100 (16)	259 (43)	247 (41)
Distant metastasis	23 (3.8)	10 (2.8)	13 (5.2)	4 (4.0)	11 (4.2)	8 (3.2)
Locoregional recurrence	14 (2.3)	8 (2.2)	6 (2.4)	1 (1.0)	8 (3.1)	5 (2.0)
Contralateral breast cancer	14 (2.3)	7 (2.0)	7 (2.8)	1 (1.0)	6 (2.3)	7 (2.8)
Death	36 (5.9)	18 (5.0)	18 (7.2)	8 (8.0)	12 (4.6)	16 (6.5)

*70-GS* 70 gene signature

### Outcome according to the 70-GS risk group

**Fig. 1:**
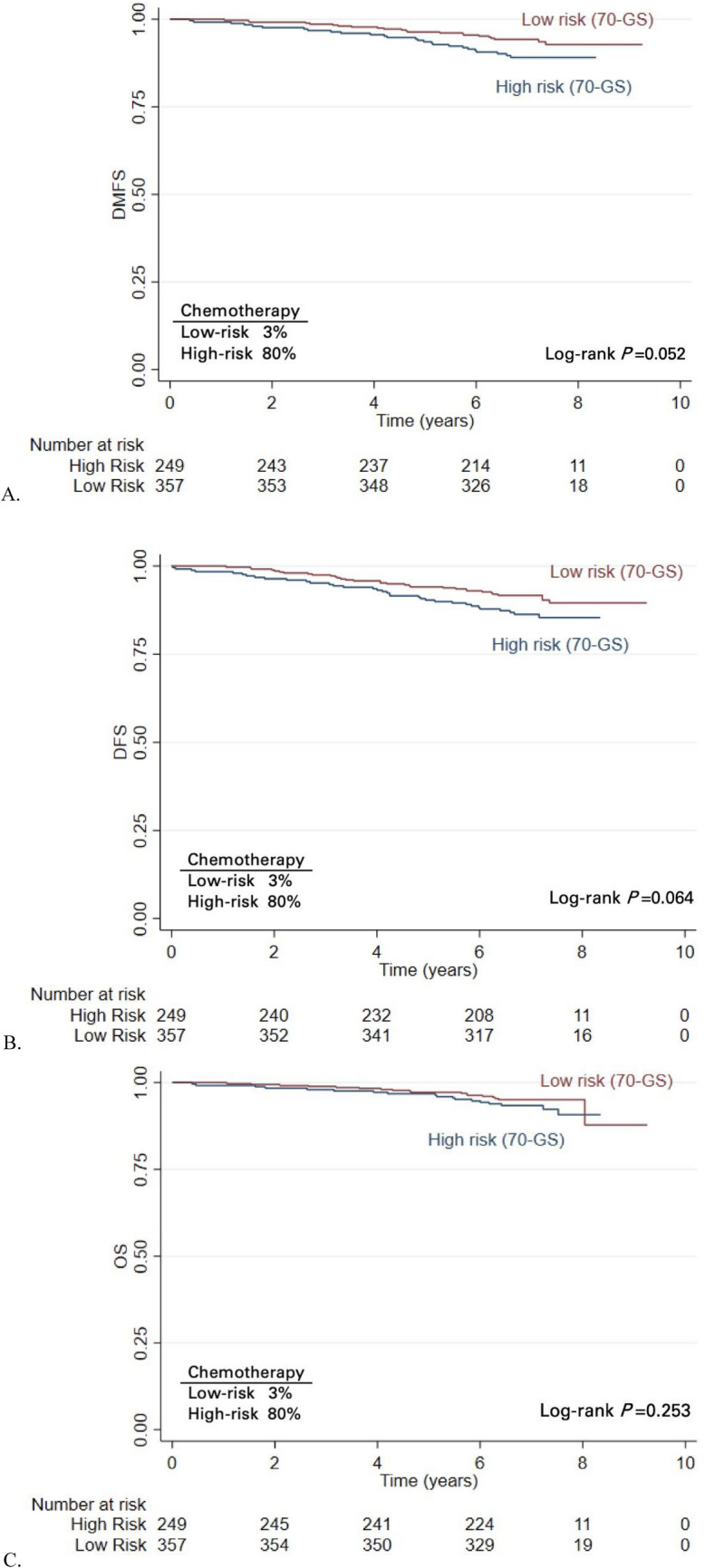
Kaplan–Meier analysis of **A** distant metastasis-free survival (DMFS), **B** disease-free survival (DFS), **C** overall survival (OS) stratified by the 70-GS genomic risk

The 7-year DMFS rate for the genomic low-risk patients, of whom 3% had received chemotherapy, was 94.2% (95% CI 91.2–96.2), while DMFS was 89.1% for the genomic high-risk patients (95% CI 84.3–92.4, *p* = 0.052), see Fig. 1.

**Fig. 2:**
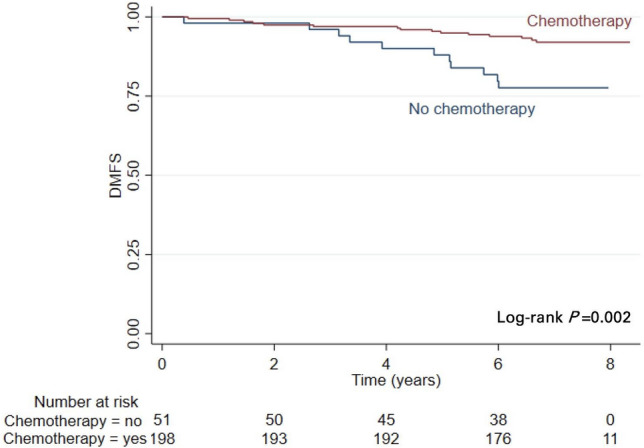
Kaplan–Meier analysis of distant metastasis-free survival (DMFS) in the 70-Gene Signature (70-GS) high-risk group in relation to the administered chemotherapy

Eighty percent of the genomic high-risk patients had been treated with adjuvant chemotherapy. Arguments to not administer adjuvant chemotherapy in 51 genomic high-risk patients were patients’ own choice (*n* = 30), oncologists’ choice (*n* = 10), reported presence of comorbidities (*n* = 3) and unknown (*n* = 8). The proportion of the genomic high-risk patients who did not receive adjuvant chemotherapy had worse DMFS than those who actually had received chemotherapy in line with the 70-GS test result: 77.6% (95% CI 63.2–86.9) versus 92.0% (95% CI 87.1–95.1), respectively (Fig. 2).

A similar trend in favor of the genomic low-risk group was observed for DFS: 7-year DFS was 91.7% (95% CI 88.3–94.2) versus 86.3% (95% CI 81.3–90.1; *p* = 0.064) for the low and high genomic risk groups, respectively. OS was nearly similar in the two groups according to the genomic risk (95.1% versus 93.4%, respectively).

### Outcome in relation to the pre-test chemotherapy advice of the oncologist before 70-GS use

**Fig. 3:**
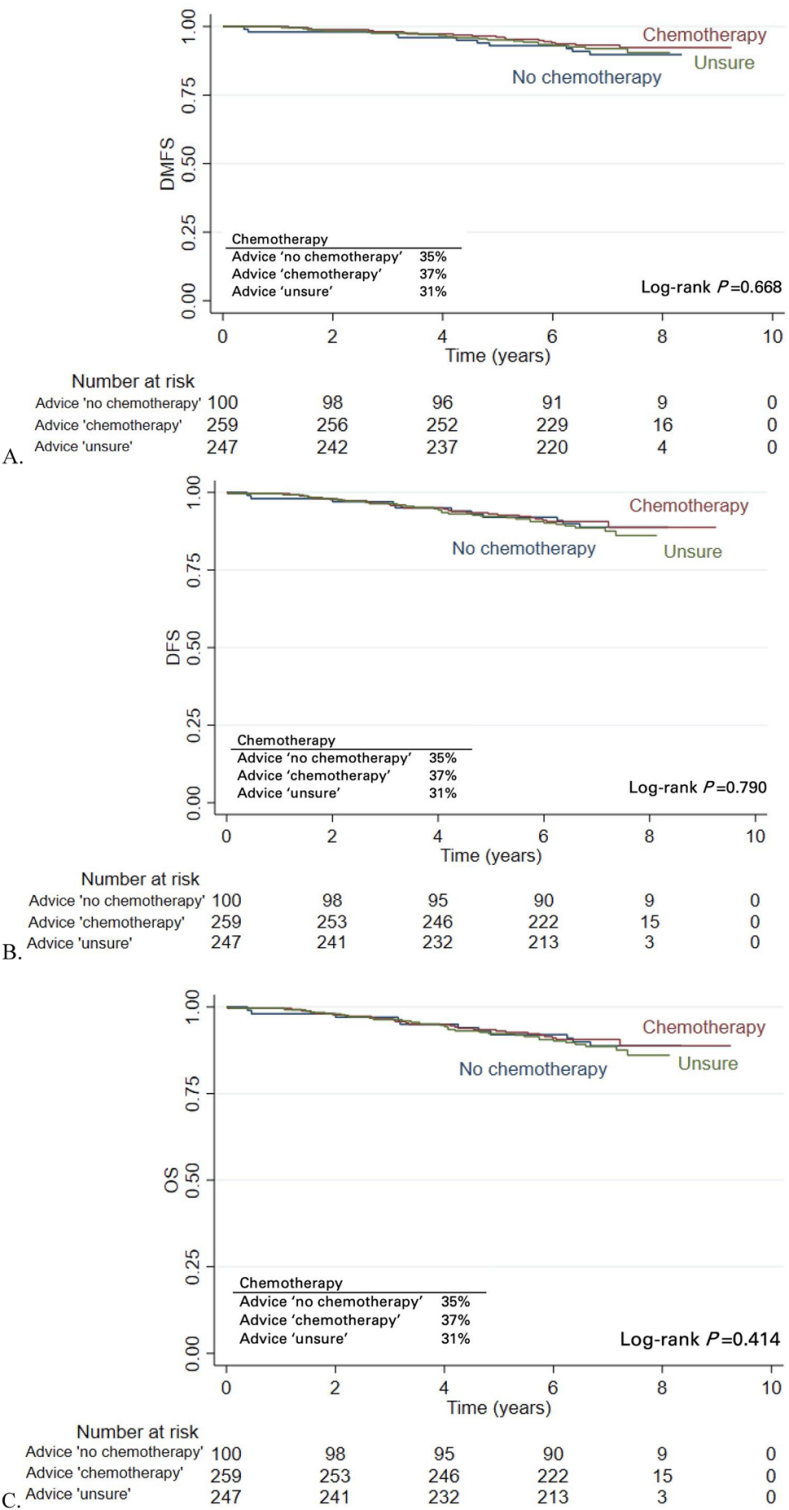
Kaplan–Meier analysis of **A** distant metastasis-free survival (DMFS), **B** disease-free survival (DFS), **C** overall survival (OS) stratified by the oncologists’ pre-test chemotherapy advice

In an exploratory analysis of outcomes in relation to the oncologists’ advice to administer chemotherapy without the 70-GS test, DMFS rates were 89.8% (95% CI 81.8–94.4) for ‘no chemotherapy’ patients, 93.2% (95% CI 89.3–95.7) for ‘chemotherapy’ patients and 92.0% (95% CI 87.6–94.8) for patients in whom the oncologist was ‘unsure’ (Fig. 3).

Chemotherapy was administered to comparable proportions of the patients in the three groups: 35%, 37% and 31%, respectively. DFS and OS rates showed similar trends as the DMFS rate: patients in whom chemotherapy was not advised before deployment of the 70-GS test had, non-significant, worse outcomes than patients in whom chemotherapy was recommended.

### DMFS according to the Adjuvant! Online-based clinical risk assessment

DMFS was also investigated applying the risk categorization used in the MINDACT trial: 69% and 31% of patients were categorized as having a low or high clinical risk. This risk categorization differed significantly from the 70-GS test result and the oncologists’ preliminary advice to administer chemotherapy without using the 70-GS (Supplementary Table 1).

Adjuvant chemotherapy had been administered to 31% of this clinical low-risk and to 42% of clinical high-risk patients. The DMFS rate after 7 years was 92.9% (95% CI 89.9–95.1) for clinical low-risk patients and 90.1% (95% CI 84.7–93.7) for clinical high-risk patients (*p* = 0.366). Within the Adjuvant! Online clinical low-risk group, subsequently categorizing patients according to the 70-GS test result revealed longer DMFS in the clinical low-risk/genomic low-risk group than in the clinical low-risk/genomic high-risk group: 95.2% (95% CI 91.6–97.2) versus 89.1% (95% CI 82.8–93.2). See suppl Fig. 1a and b.

## Discussion

In patients with ER+ early-stage breast cancer who were candidates for gene expression profiling because the indication to administer chemotherapy was considered disputable, DMFS was longer in the genomic low- than in the high-risk group while almost all patients in the low genomic risk group had not received chemotherapy in line with the 70-GS test result. The 7-year DMFS rate in genomic low-risk patients was 94% and 89% in genomic high-risk patients.

After 7 years of follow-up, the 94% DMFS rate in the genomic low-risk category surpassed the 10-year BCSS rate of 88% which was used in the aforementioned clinical trials to distinguish patients with a low from a high risk of developing distant metastases [11]. A limitation of this study is the unknown cause of death which made it impossible to define the primary endpoint as BCSS. Instead, we used DMFS as a proxy for BCSS because breast cancer-specific death is merely if not exclusively seen following the occurrence of DMs and GEPs have reported value in terms of predicting the risk of developing metastases [17]. The same outcome endpoint is reported by others too [11, 18] and the observed DMFS rate in the genomic low-risk patients in the present study is comparable to the reported long-term outcome of patients in the EORTC 10041/BIG3-04 (MINDACT) trial in whom chemotherapy administration was avoided based on their (low) genomic risk [11]. Notwithstanding the aforementioned considerations to use DMFS as a proxy for BCSS, it is noteworthy that the majority of the deceased patients did not have recorded DM in their medical records.

The observed five percent DMFS difference in favor of the genomic low-risk group was borderline significant, and this was corroborated by a similar trend in DFS and OS. It is noteworthy that only three percent of patients in the genomic low-risk group had received chemotherapy, whereas 80% of the patients in the genomic high-risk group had received chemotherapy. The risk-mitigating effect of chemotherapy in genomic high-risk patients conceivably skewed the outcome difference between the two groups. This is supported by the observation that patients in the genomic high-risk group who did not receive chemotherapy had the lowest DMFS (77.6%).

The exploratory analysis showed that the oncologists’ advice to administer chemotherapy prior to the 70-GS was not associated with DMFS. DMFS rates were comparable for the three groups according to the pre-test advice to administer chemotherapy and the proportions in these groups receiving chemotherapy were comparable too. These results suggest that the tendency of oncologists to advise or forego chemotherapy under the guideline-delineated circumstances to deploy a GEP did not adequately discriminate between low- and high-risk patients within the study population. DMFS was also evaluated for well-delineated clinical risk categories based on clinicopathological criteria that had been applied in the aforementioned clinical trials [11, 12]. This clinical risk categorization was based on the Adjuvant! Online predicted 10-year BCSS (below or above 88% percent). It was remarkable that in our study the pre-test oncologists’ advice to administer chemotherapy differed significantly from the clinical high or low risk as defined by Adjuvant! Online. The observation that the Adjuvant! Online clinical low/high-risk categorization correlates poorly with the 70-GS test result is known and has been reported by others [11, 19, 20]. Evaluating outcome for clinical high- and clinical low-risk patients revealed a trend toward longer DMFS in low clinical risk patients, yet within the clinical low-risk category the 70-GS further distinguished genomic low-risk patients who had a statistically significant longer DMFS than genomic high-risk patients in the clinical low-risk category. This implies that incorporating genomic risk into clinical prediction tools can optimize the determination of the patient’s prognosis. Several research groups are working on this subject. An example of such a prediction tool combining a patients’ clinical risk with genomic risk is the RSCIin tool [21]. It is of note that in the MINDACT trial no advantage of following the 70-GS test result, i.e., treatment with adjuvant chemotherapy, was seen in clinical low-/genomic high-risk patients.

Since the present study was initiated, the use of GEP have become routine practice albeit that the indications for GEP use have been adjusted. The former was based on the results of the aforementioned RCTs (MINDACT, TailorX). The results of the both studies propagate adhering to a GEP test result in clinical high-risk patients with a genomic low risk. More recently, the use of GEPs is being discommended in younger patients by ASCO- and other national guidelines following the results of the TAILORx- and RxPONDER trial and an update of the MINDACT trial that reported a 5% benefit of adjuvant chemotherapy in patients aged ≤ 50 years regardless of GEPs results [12, 18, 22–24]. In the Netherlands reimbursement of the 70-GS (and Oncotype DX) tests recently got secured by the health insurance companies for early-stage breast cancer patients above the age of 50 [25].

Overall, chemotherapy administration in patients with ER+ early-stage breast cancer diminished in recent years, irrespective of deployment of the 70-GS, in particular in patients without lymph node metastases (pN0) [26]. We previously reported on this decrease in chemotherapy use in the group of patients with pN0 low- and intermediate-grade luminal type breast cancers [24, 26]. In that perspective deployment of the 70-GS should be reserved for patients with more high-risk clinicopathological features such as node-positive disease with 1–3 positive nodes [22]. This shift toward use of GEPs in patients with more extensive disease is also illustrated by more recent studies and guidelines [22, 24, 27]. Then again, in our original study, we demonstrated that using the 70-GS not only led to less patients receiving chemotherapy, but also to different patients receiving chemotherapy [13]. The subsequent observations in the present study that DMFS did not differ between the oncologists’ pre-test advice risk categories and that the 70-GS was associated with DMFS within the Adjuvant! Online-based clinical low-risk category raises the question whether omitting chemotherapy without using a GEP is sensible in patients who have tumors with characteristics that leave doubt regarding the indication regarding chemotherapy.

A strength of this study is that it is multicentered and includes more than 600 patients treated in 33 hospitals. The study provides prospective real-world data regarding the long-term effect of GEP use. It presents follow-up data of a prospective study originally designed to evaluate the impact of 70-GS use on chemotherapy administration in patients in whom doubt existed regarding the beneficial effect of chemotherapy. We chose to perform separate log-rank analyses to evaluate the impact of GEP use and to address the hypothetical situation in which a GEP had not been available and the preliminary advice would have been adhered to. We deemed it inappropriate to do a multivariable analysis. The median follow-up duration of 7 years provides substantial outcome information, but longer follow-up duration remains preferable in patients with luminal breast cancers, as in this patient group recurrences are known to occur well beyond the timeframe of this study [28]. Of approximately 10 percent of patients from the original study cohort, no outcome data were retrievable for the reasons that are mentioned in the methods section. Nevertheless, patient-, tumor- and treatment-related characteristics of the current study cohort are comparable to the original study population: in both study populations 73% of the patients’ tumors was categorized as grade 2, 81% as stage T1 and 84% of the patients had no lymph node involvement. This suggests that this loss to follow-up on outcome data is random—and therefore without significant impact on results [13]. While administration of chemotherapy was recorded in the original study and verified by the NCR registrars for the present study, information about the administered chemotherapy agents is not available. National guidelines at the time uniformly advised six cycles of anthracycline containing chemotherapy regimens for ER+ breast cancer patients [7]. Furthermore, the use of DMFS as primary endpoint is disputable since death was not-breast-cancer-related in the majority of deceased patients and more than half of the patients with recorded DMs were alive at the end of the study. Another limitation of this study is the clinical risk categorization used in the exploratory analysis that was based on Adjuvant! Online criteria. While this tool was used by oncologists at the time of inclusion and used in the EORTC 10041/BIG3-04 (MIDNACT) trial, at present Adjuvant! Online is no longer available and is replaced by alternative risk assessment tools such as PREDICT [29].

In conclusion, this prospective observational study showed that the 70-GS distinguished a group of low-risk patients who did not receive chemotherapy. This group of patients had a better outcome than a group of high-risk patients of whom the majority did receive chemotherapy. As such the 70-GS better distinguished patients who did benefit from adjuvant chemotherapy than the oncologists’ pre-test advice without a GEP. It is therefore advised to deploy the 70-GS to better select patients for adjuvant chemotherapy. Further studies should provide longer and thereby more robust follow-up data and need to address the optimal interplay between clinical and genomic risk to better select patients who benefit from 70-GS use.

## Supplementary Information

Below is the link to the electronic supplementary material.

Supplementary file1 (DOCX 17 KB) Baseline table according to clinical risk based on Adjuvant! Online (concordance between the different risk stratifications)

Supplementary file2 (JPG 151 KB) Supplementary Fig. 1 Kaplan-Meier analysis of distant metastasis-free survival (DMFS) in **A** Adjuvant! Online clinical low-risk patients, **B** Adjuvant! Online clinical high-risk patients according to the 70-GS test results. Estimates are reported at 7 years because at that time point there were still a sufficient number of patients at risk. 70-GS 70 Gene Signature

Supplementary file3 (JPG 137 KB)

## Data Availability

Datasets described and analyzed in this manuscript are available from the corresponding author on reasonable request.
