# Supplementary material for: 70-Gene signature-guided adjuvant systemic treatment adjustments in early-stage ER+ breast cancer patients: 7-year follow-up of a prospective multicenter cohort study
Source: Breast Cancer Res Treat. 2024 Sep 30;209(2):331–40. doi: 10.1007/s10549-024-07496-3 (PMC11785635; doi:10.1007/s10549-024-07496-3)
Supplement: Supplementary file 1 — Supplementary file1 (DOCX 17 KB) Baseline table according to clinical risk based on Adjuvant! Online (concordance between the different risk stratifications) [file 10549_2024_7496_MOESM1_ESM.docx]

**Supplementary table 1**

***Baseline table according to clinical risk based on Adjuvant! Online***

*Concordance between the different risk stratifications*

| **Characteristics** | **N** | **Adjuvant! Online based clinical risk^a^ (N = 599^b^)** | | |
| --- | --- | --- | --- | --- |
|  |  | Low risk (%) | High risk (%) | *p* value*^c^* |
| No. of patients | 599 | 412 | 187 |  |
| **Age group (years)** |  |  |  |  |
| <40 | 8 (1) | 5 (1) | 3 (2) | *0.213* |
| 40-49 | 116 (19) | 79 (19) | 37 (20) |  |
| 50-59 | 228 (38) | 167 (41) | 61 (33) |  |
| 60-69 | 224 (38) | 149 (36) | 75 (40) |  |
| 70-79 | 22 (4) | 12 (3) | 10 (5) |  |
| >80 | 1 (0) | 0 (0) | 1 (1) |  |
| **Surgery type** |  |  |  |  |
| Lumpectomy | 485 (81) | 338 (82) | 147 (79) | *0.322* |
| Mastectomy | 114 (19) | 74 (18) | 40 (21) |  |
| **Type of axillary surgery** |  |  |  |  |
| ALND | 6 (1) | 4 (1) | 2 (1) | *0.337* |
| SNP | 566 (95) | 389 (94) | 177 (95) |  |
| ALND+SNP | 7 (1) | 3 (1) | 4 (2) |  |
| None | 20 (3) | 16 (4) | 4 (2) |  |
| **PR status** |  |  |  |  |
| Negative | 76 (13) | 51 (13) | 25 (13) | *0.755* |
| Positive | 522 (87) | 360 (87) | 162 (87) |  |
| Unknown | 1 (0) | 1 (0) | 0 (0) |  |
| **T-stage** |  |  |  |  |
| T1 | 487 (81) | 389 (95) | 98 (52) | *<0.001* |
| T2 | 111 (19) | 22 (5) | 89 (48) |  |
| T3 | 1 (0) | 1 (0) | 0 (0) |  |
| **Adjuvant chemotherapy** |  |  |  |  |
| No | 392 (65) | 284 (69) | 108 (58) | 0.008 |
| Yes | 207 (35) | 128 (31) | 79 (42) |  |
| **Adjuvant hormonal therapy** |  |  |  |  |
| No | 110 (18) | 84 (20) | 26 (14) | 0.058 |
| Yes | 489 (82) | 328 (80) | 161 (86) |  |
| **70-GS** |  |  |  |  |
| Low risk | 351 (59) | 258 (63) | 93 (50) | 0.003 |
| High risk | 248 (41) | 154 (37) | 93 (50) |  |
| **Pre-test chemotherapy advice** |  |  |  |  |
| No chemotherapy | 98 (16) | 87 (21) | 11 (6) | <0.001 |
| Chemotherapy | 259 (43) | 158 (38) | 101 (54) |  |
| Unsure | 242 (41) | 167 (41) | 75 (40) |  |

*70-GS* 70 Gene Signature, *ALND* axillary lymph node dissection, *SNP* sentinel node procedure, *ER*  estrogen receptor, *PR* progesteron receptor

^a^ Adjuvant! Online clinical risk is determined by the following: estrogen receptor status, human epidermal growth factor receptor 2 (HER2), histological grade, nodal status and tumor size.

^b^ Seven patients could not be included in this clinical risk stratification due to lack of information needed to calculate the Adjuvant! Online risk, and 1 ER+ patient.

^c^ Chi-squared test
