# Supplementary figures and images for: 70-Gene signature-guided adjuvant systemic treatment adjustments in early-stage ER+ breast cancer patients: 7-year follow-up of a prospective multicenter cohort study
Source: Breast Cancer Res Treat. 2024 Sep 30;209(2):331–40. doi: 10.1007/s10549-024-07496-3 (PMC11785635; doi:10.1007/s10549-024-07496-3)

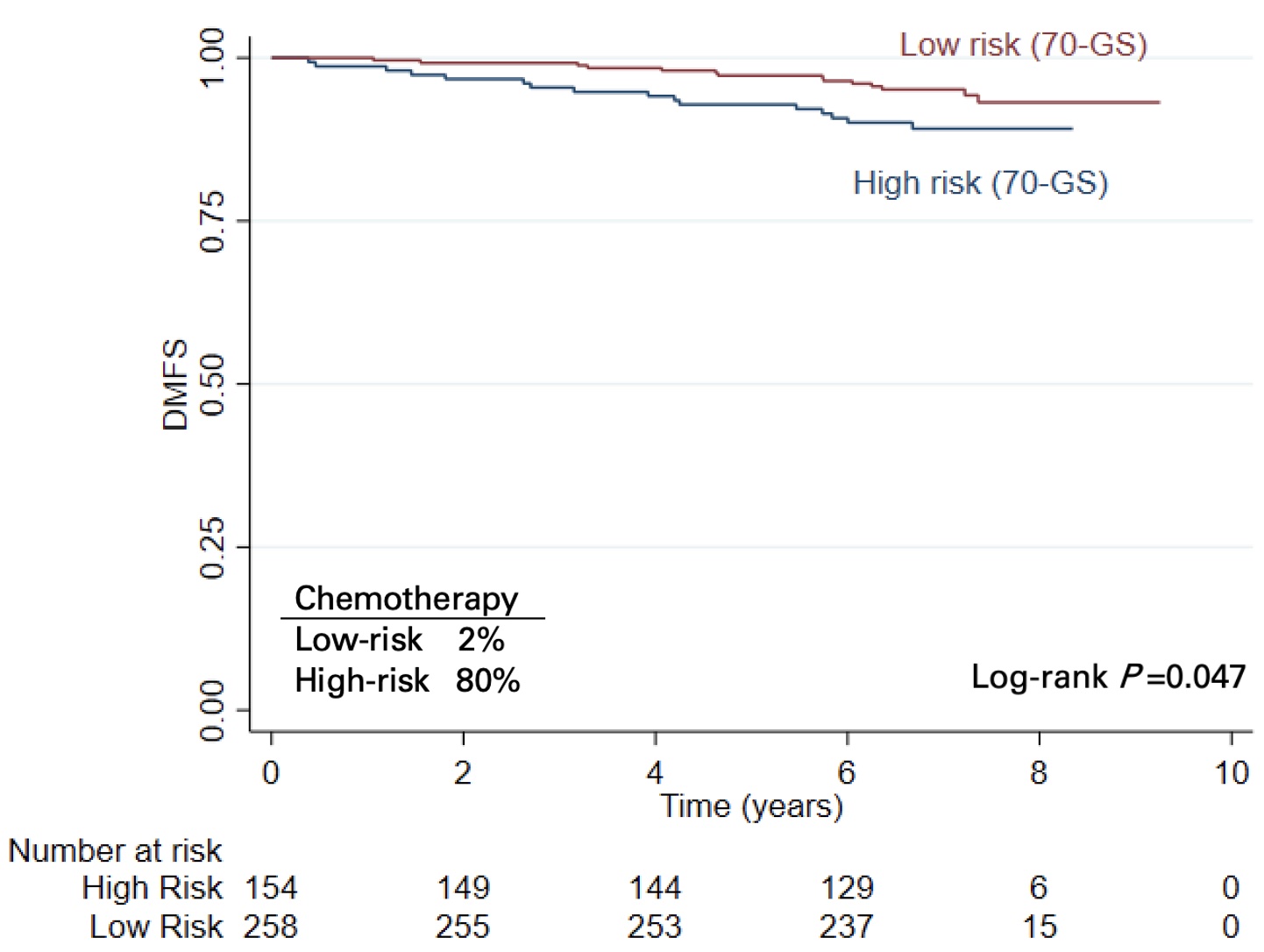

Supplement: Supplementary file 2 — Supplementary file2 (JPG 151 KB) Supplementary Fig. 1 Kaplan-Meier analysis of distant metastasis-free survival (DMFS) in A Adjuvant! Online clinical low-risk patients, B Adjuvant! Online clinical high-risk patients according to the 70-GS test results. Estimates are reported at 7 years because at that time point there were still a sufficient number of patients at risk. 70-GS 70 Gene Signature [file 10549_2024_7496_MOESM2_ESM.jpg]

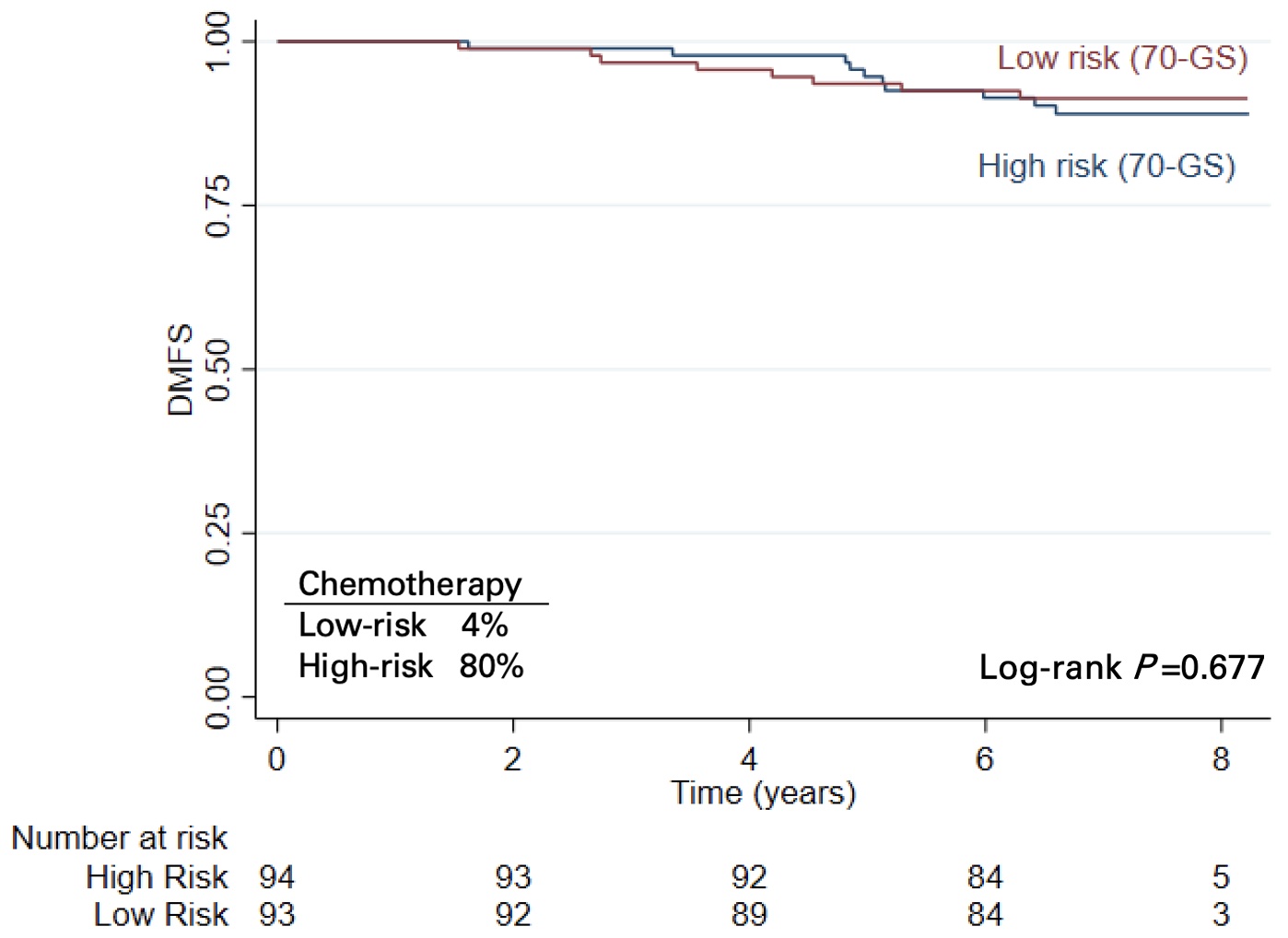

Supplement: Supplementary file 3 — Supplementary file3 (JPG 137 KB) [file 10549_2024_7496_MOESM3_ESM.jpg]
